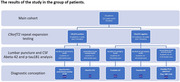# Challenges in the identification of underlying pathologies in dementias with predominant behavioral phenotype using laboratory biomarkers

**DOI:** 10.1002/alz70856_106350

**Published:** 2026-01-08

**Authors:** Kseniya V. Nevzorova, Yuliya A. Shpilyukova, Alla A. Shabalina, Natalya Yu. Abramycheva, Lusine A. Brsikyan, Ekaterina Yu. Fedotova, Sergey N. Illarioshkin

**Affiliations:** ^1^ Research Center of Neurology, Moscow, Russian Federation; ^2^ Research Center of Neurology, Moscow, Other, Russian Federation

## Abstract

**Background:**

Predominant behavioral forms of dementias are usually associated with frontotemporal lobar degeneration (FTLD) spectrum and considerably less often with atypical Alzheimer's disease (AD). The most common and clinically available genetic biomarker of FTLD is a repeat expansion in *C9orf72*, which is associated with TDP‐43 pathology. Biomarkers of Alzheimer pathology in accordance with the IWG recommendations (Dubois et al., 2021) are Abeta‐42 and *p*‐tau181 in cerebrospinal fluid (CSF). The aim of our study was to evaluate these biomarkers in a cohort of patients with predominant behavioral symptoms.

**Method:**

We examined 20 patients (9 females and 11 males, median age 64.5 [58; 72] years, median disease duration 4 [2; 5] years) with predominant behavioral impairments meeting clinical criteria of possible/probable behavioral variant of frontotemporal demetia (bvFTD) (Rascovsky et al., 2011). Positive family history of cognitive impairments was detected in 11 cases (55%). All patients underwent blood sampling for DNA testing of *C9orf72* repeat expansion (repeat‐primed PCR), and lumbar puncture for assessment of Abeta‐42 and *p*‐tau181 biomarkers in CSF (ELISA).

**Result:**

We found 3 patients (15%) with hexanucleotide repeat expansions in *C9orf72*, which allowed us to diagnose bvFTD with definite FTLD‐TDP pathology. Two of these patients (10%) additionally had a low level of CSF Abeta‐42 assuming a verified overlap of AD and FTLD‐TDP pathologies.

Among *C9orf72*‐negative patients positive biomarkers of AD pathology was detected in 8 cases (40% of all group) including 4 patients with probable AD diagnosis and 4 with possible AD. As a result in the whole cohort we found 50% cases with AD pathology, which could coexist with FTLD pathology or could be possibly isolated.

Nine patients (45%) had no pathological AD changes in CSF and no *C9orf72* repeat expansion. In these cases underlying pathologies could belong to a classic FTLD spectrum including tau, TDP, FUS.

**Conclusion:**

Neurodegenerative dementias with behavioral clinical presentation are a diverse group of diseases. In this study we showed the presence of not uncommon cases with overlapping pathologies. This highlights the importance and the need of a wide variety of laboratory tests available for clinical use in order to accurately predict in vivo underlying pathologies.